# Identification of resistance loci against new pathotypes of *Plasmodiophora brassicae* in *Brassica napus* based on genome-wide association mapping

**DOI:** 10.1038/s41598-021-85836-9

**Published:** 2021-03-23

**Authors:** Abdulsalam Dakouri, Mebarek Lamara, Md. Masud Karim, Jinghe Wang, Qilin Chen, Bruce D. Gossen, Stephen E. Strelkov, Sheau-Fang Hwang, Gary Peng, Fengqun Yu

**Affiliations:** 1 Saskatoon Research and Development Centre, Agriculture and Agri-Food Canada, Saskatoon, Canada; 2 Institut de Recherche Sur Les Forêts (IRF), Université du Québec en Abitibi-Témiscamingue, 445 boul. de l’Université, Rouyn-Noranda, QC J9X 5E4 Canada; 3 Department of Agricultural, Food and Nutritional Science, University of Alberta, Alberta, Canada

**Keywords:** Plant genetics, Plant stress responses

## Abstract

Genetic resistance is a successful strategy for management of clubroot (*Plasmodiophora brassicae*) of brassica crops, but resistance can break down quickly. Identification of novel sources of resistance is especially important when new pathotypes arise. In the current study, the reaction of 177 accessions of *Brassica napus* to four new, virulent pathotypes of *P.* *brassicae* was assessed. Each accession was genotyped using genotyping by sequencing to identify and map novel sources of clubroot resistance using mixed linear model (MLM) analysis. The majority of accessions were highly susceptible (70–100 DSI), but a few accessions exhibited strong resistance (0–20 DSI) to pathotypes 5X (21 accessions), 3A (8), 2B (7), and 3D (15), based on the Canadian Clubroot Differential system. In total, 301,753 SNPs were mapped to 19 chromosomes. Population structure analysis indicated that the 177 accessions belong to seven major populations. SNPs were associated with resistance to each pathotype using MLM. In total, 13 important SNP loci were identified, with 9 SNPs mapped to the A-genome and 4 to the C-genome. The SNPs were associated with resistance to pathotypes 5X (2 SNPs), 3A (4), 2B (5) and 3D (6). A Blast search of 1.6 Mb upstream and downstream from each SNP identified 13 disease-resistance genes or domains. The distance between a SNP locus and the nearest resistance gene ranged from 0.04 to 0.74 Mb. The resistant lines and SNP markers identified in this study can be used to breed for resistance to the most prevalent new pathotypes of *P. brassicae* in Canada.

## Introduction

Canola (*Brassica napus* L.), also known as oilseed rape, is grown around the globe. It is the largest crop in Canada, seeded on 9.1 M ha each year with a farm gate value of $16.7 billion Cdn in 2019 (www.canolacouncil.org/news/canola-council-reports-on-2019-highlighting-strength-of-value-chain-partnership/). Demand for a healthy oil for human consumption, biofuel production, and use of canola meal as a high quality feed for livestock, have produced strong prices and a steady market for canola products. Clubroot, caused by *Plasmodiophora brassicae* Woronin, is an important disease of canola and other brassica crops worldwide^1–3^. Use of clubroot-resistant (CR) canola cultivars has been the most effective and widely used strategy for clubroot management in Canada^4,5^. Management of clubroot disease using major resistance genes has been effective but not durable. For example, the resistance available in the first generation of CR canola cultivars in Canada has broken down rapidly to reveal the presence of many new pathotypes of *P. brassicae*^6,7^, which complicates breeding for resistance.

Differential systems to identify pathotypes, which consist of selected cultivars with a consistent reaction to individual pathotypes, are available for *P. brassicae*. The systems of Somé et al.^8^ and the European Clubroot Differential (ECD)^9^ are widely used in Europe, but the Williams^10^ system was selected for recommendations to producers in Canada because of its simplicity and suitability for the initial situation in Canada^8^. These three systems have recently been replaced in Canada by the Canadian Clubroot Differential (CCD) set^6^, which was designed to differentiate among the many new pathotypes recently identified in Canada. Pathotype 5X is the first new pathotype identified to be virulent on the first generation of Canadian clubroot resistant cultivars; 3A and 3D are the most common new pathotypes; and 2B is one of the most virulent new pathotypes in western Canada.

Only a couple of major clubroot resistance genes and some quantitative trait loci (QTL)^11–13^ have been identified in *B. napus* (AC genome), but strong efforts have been made to identify novel sources of resistance to clubroot in other *Brassica* spp. and transfer them into canola. The majority of genes for clubroot resistance that have been identified are from *B. rapa* subsp. *Rapifera*^9^. The resistance in *B. rapa* has been utilized successfully in breeding for resistance to clubroot in *B. napus*^5,7,8^. More than 10 major clubroot resistance genes have been identified and mapped to chromosomes of *B. rapa*^14–28^. Also, six major clubroot resistance genes and at least 10 QTL have been mapped to *B. oleracea* (C genome)^29–33^.

Genome-wide association studies (GWAS) provide a quick and precise approach for linkage mapping in QTL detection studies. GWAS uses the linkage disequilibrium (LD) between alleles within diverse populations to detect potential association between markers and the traits of interest. In the first application of GWAS to identify resistance to clubroot, a 60 K SNP array was used to map 10 QTL in *B. napus*^34^. Transcriptome-based associated has also been used to identify several QTL for resistance to clubroot in *B. napus*^35^.

Next-generation sequencing can be used to identify thousands of single nucleotide polymorphism (SNP) markers and provide a dense cover of SNPs across the host genome, even in plant species with large genomes^36^. Genotyping by sequencing (GBS) provides a cost-effective approach for generating high density SNP panels in diverse accessions^37^, and GBS-association mapping has been used in many host–pathogen systems^38,39^. However, only a few genome-wide association mapping studies have been conducted in the *B. napus–P. brassicae* system^34,35^.

In the current study, a core collection of 177 accessions from a large germplasm collection of *B. napus* from around the world was selected to conduct GBS-based GWAS in the *B. napus*-*P. brassicae* system. The objective was to identify novel sources of resistance to clubroot from a core collection of *B. napus* accessions by (1) screening the collection under controlled conditions for reaction to four new pathotypes of *P. brassicae* identified on canola in Canada, (2) assessing the genetic diversity and structure of the core collection, and (3) conducting association mapping of resistance to the four pathotypes of *P. brassicae*.

## Results

### Evaluation of clubroot reaction

**Figure 1:**
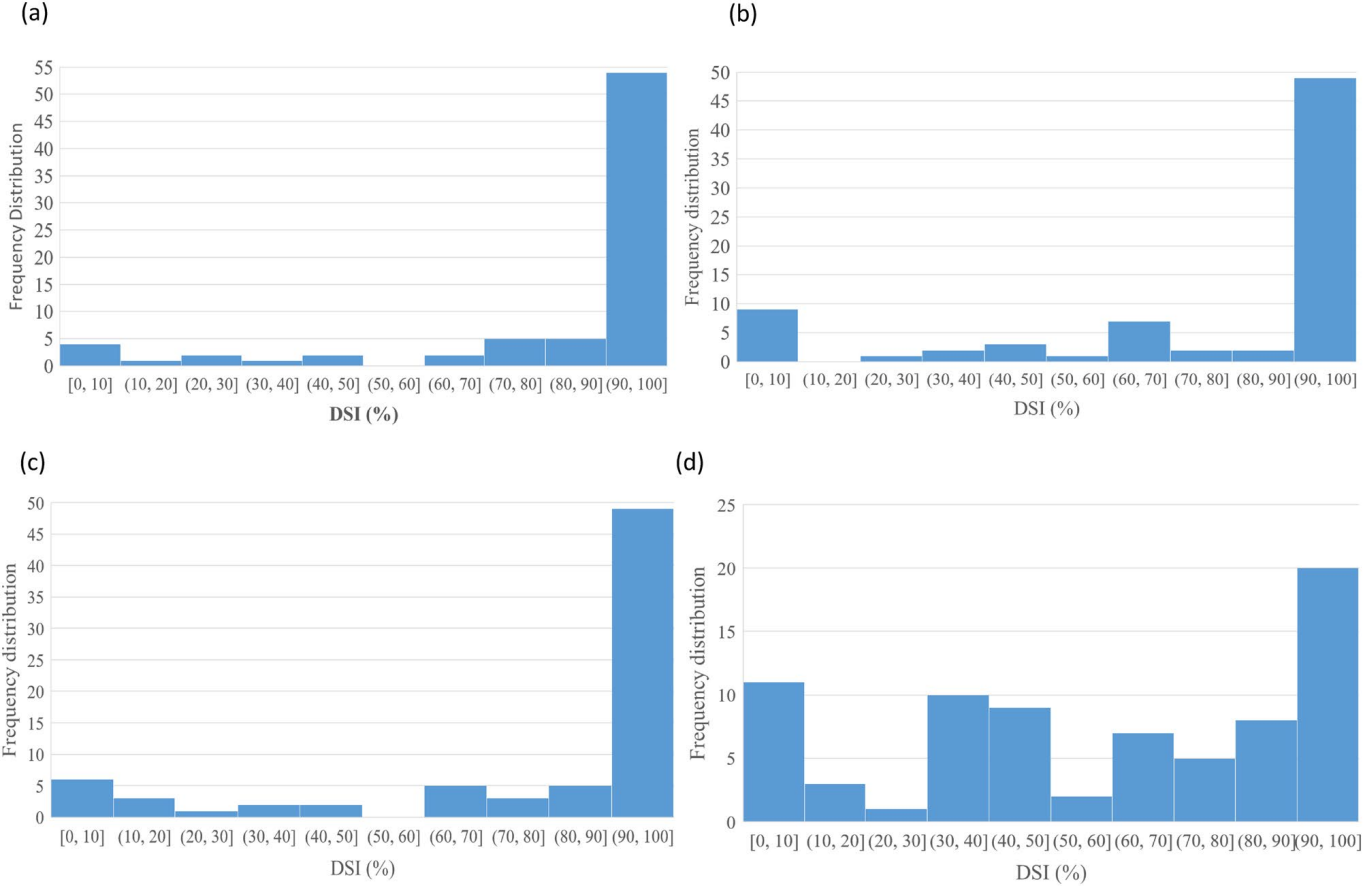
Frequency distribution of accessions of *Brassica napus* plotted against clubroot severity (disease severity index, DSI) for pathotypes (**a**) 5X, (**b**) 2B, (**c**) 3A, and (**d**) 3D.

Initially, the reaction of 671 accessions to pathotype 5X (the first pathotype identified that was virulent on the first generation of CR canola cultivars in Canada) was assessed. Only 21 accessions were resistant, defined as a disease severity index (DSI) ≤ 20. After this initial assessment had been completed, more new pathotypes were identified. The 21 accessions resistant to pathotype 5X and an additional 156 accessions were tested for resistance to the new (to western Canada) pathotypes 3A, 2B and 3D. The majority of accessions were highly susceptible (70–100 DSI), but 8 accessions were resistant to 3A (0–16 DSI), 7 accessions to 2B (0–11 DSI) and 15 accessions to 3D (0–20 DSI) (Fig. 1, Supplemental Table S1).

The resistant accessions were clustered into four groups based on their responses to combinations of pathotypes. Group 1 contained 3 accessions that were resistant to all four pathotypes. Group 2 comprised 3 accessions that were resistant to 3 of the 4 pathotypes. Group 3 had 2 accessions that were resistant to 2 out of 4 pathotypes, and Group 4 contained 25 accessions that were resistant to single pathotypes (Table 1). The majority of resistant accessions were winter oilseed rape lines of European origin. Phenotypic data were transformed using rank-based inverse normal transformation to make the DSI values fit a normal distribution, which is required for parametric model-based association analysis (Supplemental Fig. S1).

**Table 1:** Resistant accessions grouped based on their response (disease severity index) to inoculation with pathotypes 5X, 2B, 3A and 3D^6^ of *Plasmodiophora brassicae.*

Group and accession	Habit	Type	Origin	Pathotype			
				5X	2B	3A	3D
**Group-1**							
CGN06822	Winter	Oilseed rape	Europe	0	0	6	6
CGN06901	Winter	Swede rape	Europe	0	0	0	0
CGN17369	Winter	Oilseed rape	Europe	0	0	0	0
**Group-2**							
PI432393	Spring	Oilseed rape	Asia	100	0	0	0
PI443015	Winter	Oilseed rape	Europe	3	11	4	29
CGN07233	Winter	Fodder rapa	Europe	97	0	17	0
**Group-3**							
CGN15182	Winter	Fodder rapa	Europe	86	61	11	17
CGN17377	Winter	Oilseed rape	Europe	12	69	78	15
CGN06884	Winter	Oilseed rape	Europe	14	100	100	100
**Group-4**							
Ames20126	Spring	Oilseed rape	North America	100		11	
CGN06896	Winter	Oilseed rape	Europe	0	50	100	50
CGN06900	Winter	Swede rape	Europe	6	100	100	100
CGN07231	Winter	Fodder rapa	Europe	97	10	100	100
CGN14115	Winter	Oilseed rape	Europe	97	92	92	11
CGN15177	Winter	Fodder rapa	Europe	100	22	33	0
CGN17304	Winter	Oilseed rape	Europe	100	100	67	18
CGN17313	Winter	Oilseed rape	Europe	89	100	100	8
CGN17316	Winter	Oilseed rape	Europe	94	50	78	0
CGN17337	Winter	Oilseed rape	Europe	97	100	100	20
CGN17339	Winter	Oilseed rape	Europe	86	95	92	15
CGN17379	Winter	Oilseed rape	Europe	14	83	100	41
CGN17381	Winter	Oilseed rape	Europe	0	97	97	31
CGN18957	Winter	Oilseed rape	Europe	0	100	100	41
CN101875	Spring	Oilseed rape	North America	10	100	100	75
CN107671	Spring	Oilseed rape	Asia	0			
CN107681	Spring	Oilseed rape	North America	0	100	100	0
CN31149	Winter	Rutabaga	Europe	10			
CN31150	Winter	Rutabaga	Europe	5			
CN40224	Spring	Oilseed rape	Europe	0			
CN46234	Winter	Oilseed rape	North America	10			
MENDEL	Winter	Oilseed rape	Europe	0	44	31	28
Pabularia	Winter	Oilseed rape	Europe	0			
PI469890	Spring	Oilseed rape	Asia	0			

‘.’ Untested due to poor quality of seed.

### Sequence analysis and SNP discovery

GBS analysis of the 177 *B. napus* accessions generated ~ 1.2 billion total reads and ~ 633 million good barcoded reads, which were split into three FASTQ fills. On average, there were 3.3 M read counts per sample (range ~ 1.8 to 7.7 M) and 3.1 M read counts mapped (range 76–96%). Sequence tags from each file were captured and merged to produce a master tag file of 4,253,499 sequence tags. The tags were then aligned to *B. napus* reference genome v4.1 using the TASSEL-GBS pipeline. A total of 2,217,292 (52.1%) tags were uniquely aligned to the reference, 1,220,090 (28.7%) aligned to multiple positions and 816,117 (19.2%) were not aligned. Uniquely mapped tags were used to calculate the tag density distribution at each site in the *B. napus* genome and for SNP calling.

The raw sequence data for SNP calling were also analysed using the TASSEL-GBS pipeline. A total of 399,234 unfiltered SNPs and 355,680 filtered SNPs were called for the 177 accessions, with a mean individual depth of 8.5 ± 2.0 SD and mean site depth of 6.7 ± 11.4 SD. Of the 355,680 filtered SNPs, 301,753 SNPs (84.83%) were mapped to the 19 chromosomes of *B. napus* and were kept for further analyses. The remaining SNPs were randomly distributed without specific chromosome assignment, and so were discarded.

### Variant analysis and annotation

There were slightly more SNPs in the C-genome (160,174 SNPs) than the A-genome (141,579 SNPs). Chromosome A03 had the highest number of SNPs within the A-genome, while C03 contained the highest number of SNPs in the C- genome (Table 2). The mean density was 2.12 SNP/Kb across the 19 chromosomes. In general, SNP density was higher in the C-genome (2.55 SNPs/Kb) than the A-genome (1.70 SNPs/Kb). C07 had the highest number of SNPs per Kb (2.88) and A10 had the lowest (1.43) (Table 2). The vast majority of SNPs were bi-allelic (90%), and only 10% were multi-allelic (Supplemental Fig. S2). There was a positive correlation (r = 0.80) between chromosome length and the number of SNPs, but only a weak correlation (r = 0.30) between the number of SNPs and the number of SNPs per Kb.

**Table 2:** Distribution of SNPs, minor allele frequency (MAF), heterozygosity, polymorphic information content (PIC) and linkage disequilibrium (LD) on each chromosome in the A and C genomes of 117 accessions of *Brassica napus.*

Genome and Chromosome	Start	End	Total no. seq	SNP	SNP / Kb	MAF	Hetero-zygosity	PIC	Average LD
**A genome**									
A01	2024	23,251,220	23,250	13,062	1.78	0.14	0.08	0.24	0.090
A02	919	24,785,167	24,784	12,455	1.99	0.13	0.08	0.23	0.080
A03	808	29,746,073	29,745	20,541	1.45	0.14	0.07	0.24	0.060
A04	1717	19,141,470	19,140	10,562	1.81	0.14	0.07	0.24	0.090
A05	2697	23,052,978	23,050	14,917	1.55	0.14	0.06	0.24	0.076
A06	2120	24,372,251	24,370	14,696	1.66	0.13	0.06	0.22	0.075
A07	10,938	24,000,655	23,990	14,232	1.69	0.15	0.07	0.24	0.070
A08	1729	18,958,296	18,957	10,281	1.84	0.13	0.07	0.22	0.084
A09	1327	33,857,792	33,857	18,702	1.81	0.12	0.07	0.21	0.010
A10	4083	17,366,872	17,363	12,131	1.43	0.14	0.07	0.23	0.080
**Mean**			23,851	14,158	1.70	0.14	0.07	0.23	0.072
**C genome**									
C01	8039	38,812,658	38,805	17,087	2.27	0.16	0.08	0.27	0.190
C02	1607	46,186,975	46,185	17,662	2.61	0.14	0.09	0.24	0.146
C03	760	60,565,276	60,565	25,136	2.41	0.15	0.09	0.24	0.073
C04	1773	48,929,072	48,927	19,053	2.57	0.14	0.08	0.24	0.140
C05	3386	43,172,068	43,169	16,540	2.61	0.13	0.10	0.22	0.074
C06	1745	37,224,854	37,223	14,761	2.52	0.14	0.09	0.23	0.079
C07	7046	44,766,293	44,760	15,558	2.88	0.13	0.09	0.22	0.083
C08	6385	38,472,912	38,467	16,082	2.39	0.14	0.09	0.23	0.105
C09	1884	48,501,448	48,500	18,295	2.65	0.12	0.09	0.21	0.075
**Mean**			45,178	17,797	2.55	0.14	0.09	0.23	0.107
**Overall mean**			34,514	15,978	2.12	0.14	0.08	0.23	0.088

The SNPs were annotated using SnpEff software. About 37% of SNPs were annotated within coding regions, 22% within introns, 31% within promoter regions, 0.3% within splice sites, and 9.7% mapped to other genetic regions (Supplemental Fig. S3a). A more detailed SNP annotation was performed using the Variant Effect Predictor (Supplemental Fig. S3b). For SNPs within coding regions, 17% were non-synonymous, 18% were upstream-gene variants, 9% were downstream-gene variants, 23% were synonymous variants, 14% were intron variants, 15% intergenic variants, and 4% were located in the splice site regions and 5′ and 3′ UTRs (Supplemental Fig. S3b). Overall, more SNPs were annotated to the A-genome than to the C-genome (Supplemental Fig. S3c).

### Genetic diversity and population structure

For genetic diversity analysis, the SNP markers were filtered based on a minor allele frequency (MAF) of 0.05 and minimum sample count of 80%, which resulted in 104,184 high quality SNPs. The mean MAF was the same for the A- and C-genomes (MAF = 0.14). Chromosome C01 had the highest MAF (0.16), followed by C03 and A07 (0.15), and lowest MAF (0.12) was in chromosomes A09 and C09 (Table 2). The mean marker heterozygosity (H_e_) was 0.06 and the mean accession heterozygosity was 0.14. The average polymorphic information content (PIC) was the same for the A- and C-genomes (0.26). PIC was highest in chromosome C01 (0.27) and lowest (0.24) in A09 (Table 2). The ratio of transitions (changes from A <—> G and C <—> T) to transversions (changes from A <—> C, A <—> T, G <—> C or G <—> T) was 3: 2.

**Figure 2:**
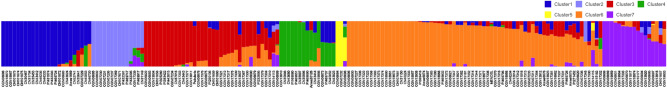
Population structure analysis of 177 accessions of *Brassica napus* based on model-based Bayesian clustering using STRUCTURE for K = 7 groups.

Population structure analysis indicated that the core collection was comprised of seven major clusters (Fig. 2, Supplemental Fig. S4). Population 1 contained 24 accessions (13.6%) representing all continents, but North American accessions were most frequently represented (10/24). Population 2 contained 14 accessions (7.9%), exclusively from Asia and Europe. Population 3 contained 36 accessions (20.3%) from all continents, but mainly from Europe. Population 4 contained 14 accessions (8.5%), mostly from Europe and Asia. Population 5 contained three accession (1.7%) from Europe and Asia. Population 6 contained 68 accessions (38.4%), mainly from Europe. Population 7 included 17 accessions (9.6%), almost exclusively from Europe (Fig. 2).

### Linkage disequilibrium analysis

Linkage disequilibrium (LD) in the association panel was calculated using Pearson’s *r*^2^ statistic on pairwise combinations of SNPs present across the 19 chromosomes of *B. napus* (Supplemental Fig. S5). The average LD (*r*^2^) across the genome was 0.15. The mean LD was 0.10 in the A-genome and 0.19 in the C-genome. LD values ranged from 0.01 in A09 to 0.19 in C01 (Table 2). Across the genome, LD decayed very rapidly (*r*^2^ = 0.23) within 1.6 Mb (Supplemental Fig. S5).

### Genome-wide association analysis

Genome-wide association analysis for clubroot severity was conducted using a general linear model (GLM) for naïve and P + Q analysis, and a mixed linear model (MLM) for P + K and P + Q + K. Naïve refers to genotypes and phenotypes only, Q is structure, and K is kinship. The quantile–quantile (Q–Q) plots from all models revealed that, save for significant SNPs, the distribution of observed – log_10_(p) was closest to the expected distribution in the P + Q + K compared to other models, so associations were identified using this model. A significance level of *P* ≤ 0.05/N (N: number of SNPs) based on the Bonferroni correction and a less stringent suggestive significance level at *P* ≤ 0.5/N were selected.

**Figure 3:**
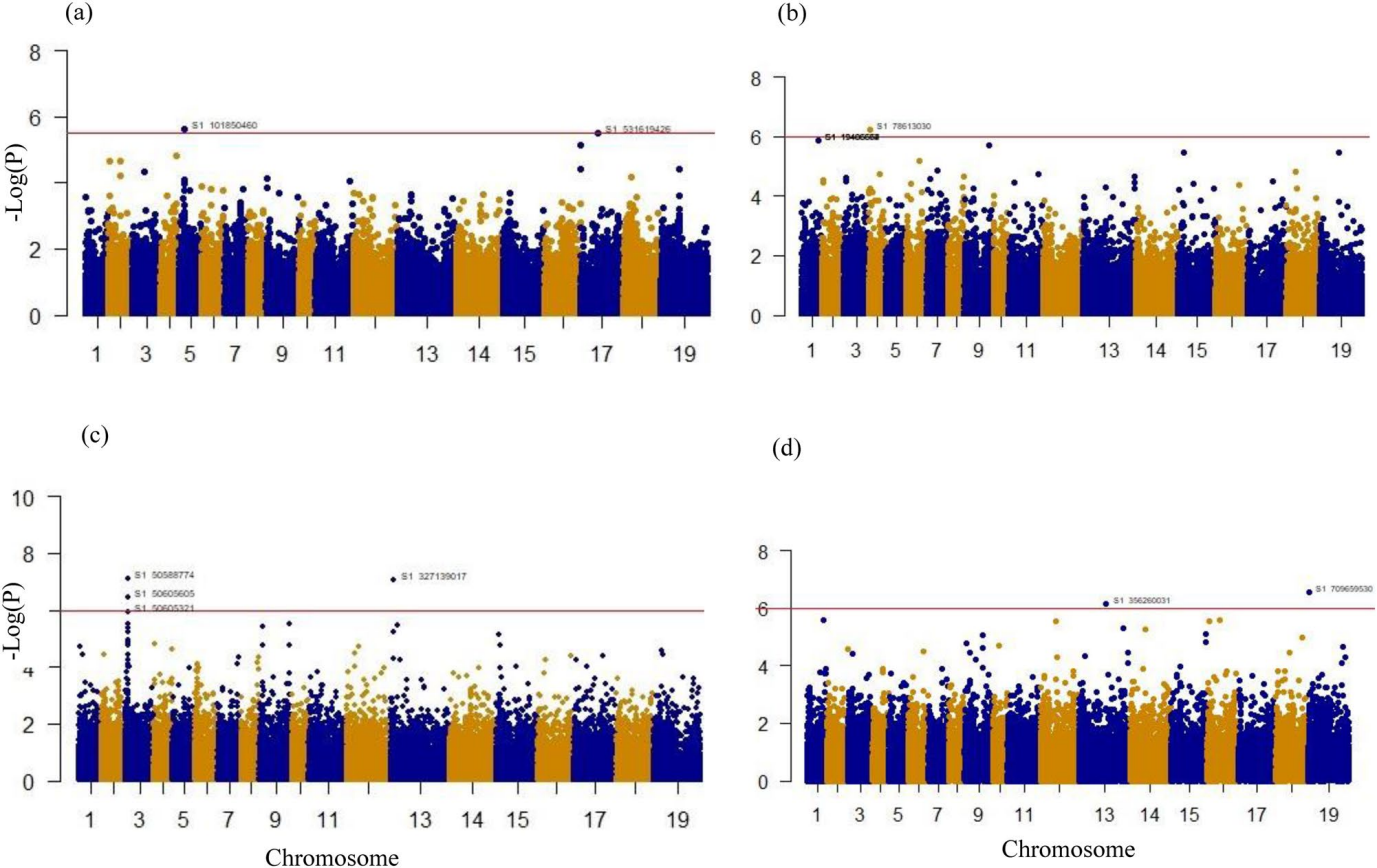
Manhattan plots of association analysis using mixed linear model (MLM) model P + K for pathotypes (**a**) 5X, (**b**) 2B, (**c**) 3A and (**d**) 3D. The horizontal line represents the threshold of significance (− log_10_0.5/104,824 SNPs = 5.32).

Association analysis detected 13 SNPs associated with resistance to the four *P. brassicae* pathotypes distributed on chromosomes A01, A03, A04, A05, C03, C07 and C09, with two SNPs (one on A05 and one on C07) associated with resistance to 5X, four SNPs to 3A (three on A03 and one on C03), three SNPs to 2B (on A01) and six SNPs (four on A01, one on C03 and one on C09) associated with resistance to 3D (Table 3, Fig. 3).

**Table 3:** List of significant SNPs, chromosomes, physical location, P values, gene ID, description, and mutation type in 117 accessions of *Brassica napus.*

Patho-type	SNP	CHR	Position	P value	Gene ID	Description	Mutation type
5X	A05_101850460	A05	4,869,317	2.38E−06	BnaA05g08830D	Protein NAP1 isoform X1	upstream gene
5X	C07_531619426	C07	17,975,139	3.01E−06	BnaC07g12510D	purple acid phosphatase 10	intergenic gene
2B	A04_78613030	A04	783,647	5.79E−07	BnaA04g01220D	Phosphotidate cytidylyltransferase 5, chloroplastic	downstream gene
2B	A01_19406654	A01	19,406,654	1.33E−06	BnaA01g27830D	Glutamate decarboxylase 5	synonymous
2B	A01_19406667	A01	19,406,667	1.33E−06	BnaA01g27830D	Glutamate decarboxylase 5	missense
2B	A01_19406668	A01	19,406,668	1.33E−06	BnaA01g27830D	Glutamate decarboxylase 5	missense
2B	A01_19406672	A01	19,406,672	1.33E−06	BnaA01g27830D	Glutamate decarboxylase 5	synonymous
3A	A03_50588774	A03	2,526,981	7.63E−08	BnaA03g05570D	Solute carrier family 25 member 33	missense
3A	C03_327139017	C03	3,409,940	8.52E−08	BnaC03g07170D	Galactose oxidase/kelch repeat superfamily protein	synonymous
3A	A03_50605605	A03	2,543,812	3.42E−07	BnaA03g05600D	Galactose oxidase/kelch repeat superfamily protein	synonymous
3A	A03_50605321	A03	2,543,528	1.15E−06	BnaA03g05600D	Galactose oxidase/kelch repeat superfamily protein	synonymous
3D	A01_19406654	A01	19,406,654	1.33E−06	BnaA01g27830D	Glutamate decarboxylase 5	synonymous
3D	A01_19406667	A01	19,406,667	1.33E−06	BnaA01g27830D	Glutamate decarboxylase 5	missense
3D	A01_19406668	A01	19,406,668	1.33E−06	BnaA01g27830D	Glutamate decarboxylase 5	missense
3D	A01_19406672	A01	19,406,672	1.33E−06	BnaA01g27830D	Glutamate decarboxylase 5	synonymous
3D	C03_356260031	C03	32,530,954	6.83E−07	BnaC03g47440D	cysteine-rich repeat secretory-like protein (DUF26 and DUF1204)	downstream gene variant
3D	C09_709659530	C09	1,449,707	2.81E−07	BnaC09g02600D	Small nuclear ribonucleoprotein family protein	downstream gene

Four significant SNPs (A01_19406654, A01_19406667, A01_19406668 and A01_19406672) on chromosome A01 were associated with resistance to pathotypes 2B and 3D. These four SNPs were in the same *B. napus* gene, *BnaA01g27830D*, which encoded glutamate decarboxylase 5. For pathotypes 3A, two significant SNPs (A03_50605605 and A03_50605321) on chromosome A03 were detected in the same gene, *BnaA03g05600D,* and SNP C03_327139017 on chromosome C03 was detected in gene *BnaC03g07170D*. Both genes on chromosomes A03 and C03 encode galactose oxidase/kelch repeat superfamily protein (Table 3).

### Candidate resistance genes

A Blast search of 1.6 Mb upstream and downstream regions (distance based on LD) from significant SNPs was performed to identify any nucleotide-binding-site/leucine-rich-repeat (NBS-LRR) resistance protein encoding genes. For pathotype 5X, a coiled coil / nucleotide binding site / leucine-rich repeat (CC-NBS-LRR or CNL) family was identified at 0.53 Mb from the significant SNP, A05_101850460, on chromosome A05. For the SNP on C07, no NBS-LRR gene was found (Table 4). For pathotype 2B, no NBS-LRR gene was found in LD vicinity of the SNP on A04, while for SNPs on A01, a putative disease resistance protein, *At3g15700*, was detected at 0.16 Mb from the SNPs (Table 4). For pathotype 3A, no disease resistance genes were found adjacent to SNP A03_50588774 on A03, but a gene encoding an LRR domain was found at 0.08 Mb from the SNP. For SNP C03_327139017 on C03, an avirulence-induced gene (AIG1) family protein was detected at 0.04 Mb from the SNP. For pathotype 3D, a toll-interleukin-1 receptor/nucleotide binding site/leucine-rich repeat (TIR-NBS-LRR or TNL) gene was found at 0.07 Mb from the SNP, C03_356260031, on C03 (Table 4).

**Table 4:** Disease resistance genes located within candidate gene region (Mb) of SNP loci.

Pathotype & SNP	Gene ID	Description	Distance to SNP (Mb)
**5X**			
A05_101850460	BnaA05g07900D	Disease resistance protein (CC-NBS-LRR class) family	0.53
C07_531619426	None	–	–
**2B**			
A04_78613030	None	–	–
A01_19406654	BnaA01g27560D	Putative disease resistance protein At3g15700	0.16
A01_19406667	BnaA01g27560D	Putative disease resistance protein At3g15700	0.16
A01_19406668	BnaA01g27560D	Putative disease resistance protein At3g15700	0.16
A01_19406672	BnaA01g27560D	Putative disease resistance protein At3g15700	0.16
**3A**			
A03_50588774	BnaA03g065730	F-Box LRR repeat protein At2g430260-like	0.08
C03_327139017	BnaC03g07250D	Avirulence induced gene (AIG1) family protein	0.04
A03_50605605	BnaA03g03830D	disease resistance protein TAO1-like	0.74
A03_50605321	BnaA03g03830D	disease resistance protein TAO1-like	0.74
**3D**			
C09_709659530	BnaC09g02130D	probable disease resistance protein At1g59620	0.31
C03_356260031	BnaC03g47400D	Disease resistance protein (TIR-NBS-LRR class) family	0.07
A01_19406654	BnaA01g27560D	Putative disease resistance protein At3g15700	0.16
A01_19406667	BnaA01g27560D	Putative disease resistance protein At3g15700	0.16
A01_19406668	BnaA01g27560D	Putative disease resistance protein At3g15700	0.16
A01_19406672	BnaA01g27560D	Putative disease resistance protein At3g15700	0.16

## Discussion

In the current study, GWAS was used to identify and map new sources of resistance to four recently identified pathotypes (5X, 3A, 2B and 3D) of *P. brassicae* in 177 accessions of *B. napus* from around the world. The majority of the accessions were highly susceptible to all four pathotypes (80–100 DSI), but ~ 10% showed strong resistance (0–20 DSI). This indicated that sources of strong resistance to clubroot were much less common in *B. napus* than in *B. rapa*^24,25^. In total, 13 SNPs were associated with resistance, of which nine SNPs were on the A-genome, and four on the C-genome. Although the A-genome (from *B. rapa*) appeared to carry more QTL for clubroot resistance than the C-genome, this result indicated that the C-genome (from *B. oleracea*) could also be a potential source for clubroot resistance. Interestingly, four SNPs on A01 were common for pathotypes 2B and 3D, which may indicate that there is a common QTL for resistance to the two pathotypes. Moreover, SNPs located on A03 and C03 were associated with resistance to pathotype 3A. A Blast search determined that these SNPs were located very close to or within the same gene on both chromosomes. Association of SNPs with a single gene on separate chromosomes indicated the occurrence of inter-genome gene duplication, which is a common phenomenon in *B. napus*^40–42^.

QTL for clubroot resistance have been identified previously in *B. napus*, and several have been mapped to chromosomes A01, A02, A03, A08, C02, C03, C06, C07, and C09^34,35^. In those previous studies, the disease reaction to only a single strain of *P. brassicae* was assessed, and a SNP array or associative transcriptome was used rather than GBS, which might explain the difference in the number and identity of QTL detected. In a recent GWAS study, 45 QTLs were identified against 13 strains on chromosomes A01 to A10, and C02, C03 and C05^43^. In the current study, two pathotypes (3A and 2B) included in a recent study^43^ were analysed using a larger and potentially more diverse germplasm collection. The current study, however, identified different QTL from those in the previous study^43^. One possible explanation for the difference between the studies could be the analysis of DSI. A rank-based inverse normal transformation was used to make the DSI values nearly fit the normal distribution required for parametric model-based association analysis in the current study, while highly skewed DSIs were used in the previous study^43^. In addition, different sources of *B. napus* collections in the current study from the previous report^43^ could be an important factor contributing to the discrepancy. We conclude that all 13 QTL identified in the current study are likely to be novel because they were located at different physical locations on the chromosomes from the QTL identified previously^43^.

Extensive efforts have been made to map the genes for resistance to *P. brassicae* in *Brassica* species via bi-parental mapping approaches. In our previous studies, eight genes / QTL loci for resistance to pathotype 3H or 5X or both were mapped into chromosomes A02 (*Rcr8*), A03 (*Rcr1*, *Rcr2*, *Rcr4* and *Rcr5*) and A08 (*Rcr3*, *Rcr9* and *Rcr9*^*wa*^), and one gene into chromosome C07 (*Rcr7*)^13,14,22–25^. In the current study, 13 SNPs were identified from seven chromosomes (A01, A03, A04, A05, C03, C07 and C09) for resistance to four new pathotypes (2B, 3A, 3D and 5X) of *P. brassica* from Canada. None of the previously identified genes/QTL for resistance to Canadian pathotypes reside in chromosomes A01, A04, A05, C03 or C09. Although three SNPs were identified in chromosome A03 where *Rcr1, Rcr2, Rcr4* and *Rcr5* were located and one SNP in chromosome C07 where *Rcr7* was located, these four SNPs were located in different regions from the identified genes. Three closely linked SNP A03_50588774, A03_50605321 and A03_50605605 associated with resistance to 3A were identified in the 2.5 Mb region of chromosome A03, while *Rcr1*, *Rcr2*, *Rcr4* and *Rcr5* spanned in the region of 23–25 Mb of chromosome A03 in the *B. napus* reference genome. Furthermore, SNP C07_531619426 associated with resistance to 5X was located at 17.9 Mb of chromosome C07. However, *Rcr7* was identified in *B. oleraces*, in a region equivalent to the 25 Mb of chromosome C07 in the *B. napus* genome.

The majority of disease resistance genes identified in plants have been classified as TNL or CNL proteins, with ~ 70% of NBS-LRR genes in the Brassicaceae family being TNLs^44–47^. In the current study, five putative resistance genes were identified within LD distance of the SNPs associated with resistance to the four pathotypes. The resistance genes belonged to the TNL family (one gene) and the CNL family (one gene), as well as other types of resistance genes. The gene *BnaA03g065730,* which mapped close to SNP A03_50588774, contained the LRR protein domain that is an essential part of NBS-LRR proteins^48^. Moreover, the gene *BnaC03g07250D* that was located at 0.04 Mb from SNP C03_327139017 encoded an avirulence-induced gene (AIG1) family protein that, according to the gene-for-gene hypothesis by Flor^49^, should belong to a disease resistance protein-encoding gene family.

Population structure can have a significant impact on GWAS. Structure analysis using the entire SNP panel from GBS indicated that the core collection was comprised of seven sub-populations. However, a previous study using a SNP panel obtained from the Brassica 60 K Illumina Infinium SNP array identified only two major subpopulation^50^. Similarly, the difference in LD between studies at the chromosomal level as well as at the genome level appeared to be associated with the density of molecular markers. In the current study, LD extended further in the C-genome relative to the A-genome. LD decayed very rapidly (*r*^2^ = 0.23) within 1.6 Mb, while the range of previous reported LD decay values was affected by the diversity within the germplasm collection that was examined^51–53^.

In summary, the current study identified several accessions of *B. napus* with high levels of resistance to one or more or the four important new pathotypes of *P. brassicae* examined. Genome-wide association mapping analysis detected and mapped 13 SNP loci associated with resistance to the four pathotypes. This information will be used in subsequent genetic analysis of bi-parental populations to verify the SNPs and fine map the functional genes responsible for resistance to each pathotype, and also used for marker-assisted breeding of resistance to clubroot in canola.

## Materials and methods

### Plant and pathogen materials

A collection of 671 *B. napus* accessions were obtained from three gene banks; the Plant Genetic Resources of Canada, the Centre for Genetic Resources of the Netherlands, and Agricultural Research Service of the United of America. Self-pollination was performed for each line under greenhouse conditions to reduce the level of heterozygosity. The accessions were evaluated for resistance to a field collection of pathotype 5X (strain LG02) that had been characterized by Dr. S.E. Strelkov using the Canadian Clubroot Differential^6^ system. Also, selected lines were tested for resistance to field collections of pathotypes 3A (strain F.P. 3–14), 2B (F.P. 183–14) and 3D (F.P. 1–14) of *P. brassicae* (also provided by Dr. Strelkov) and then genotyped using a GBS platform.

A core group of 177 accessions from 32 countries was selected for GWAS. This group included accessions of oilseed rape (146 accessions), fodder rape (21), Swede rape (7), rutabaga (2) and turnip (1) from Europe (123 accessions), Asia (29), North America (20), Oceania (2), South America (1), Africa (1), and one accession of unknown origin (Supplemental Table S1). The growth habit was predominantly winter type (129), with some spring types (48) (Supplemental Table S1).

Seedlings for GBS analysis were grown to the 3–4 leaf stage in a growth chamber. A small (100 mg) sample of leaf tissue was collected from each accession, immediately frozen in liquid nitrogen, lyophilized in a freeze dryer for ~ 48 h and ground to a fine powder using a tissue lyser (Qiagen, Newtown City, USA).

Resting spores of pathotypes 5X, 3A, 2B, and 3D of *P. brassicae* were increased on susceptible canola and stored as frozen clubbed roots at − 20 °C until needed. Resting spores were extracted from the frozen clubs as described by Strelkov et al.^54^, and adjusted to a concentration of 1 × 10^7^ resting spores mL^−1^. Spores of each pathotype were applied separately to the host entries.

### Evaluation of clubroot reaction

The experiment was conducted in a growth chamber under the controlled environment following the method similar to that described by Suwabe et al.^16^. Seed of each host genotype was pre-germinated on moistened filter paper in a Petri dish. One-week-old seedlings of each host line × pathotype combination with 12 plants each were inoculated by dipping the entire root system in the resting spore suspension for 10 s. The inoculated seedlings were then immediately planted in 6 cm × 6 cm × 6 cm plastic pots filled with Sunshine LA4 potting mixture, with one seedling per pot. The pots were thoroughly watered and transferred to a greenhouse at 21 °C ± 2 °C with a 16 h photoperiod. The potting mixture was kept saturated with tap water at pH 6.5 for the first week after inoculation and then watered and fertilized as required.

Six weeks after inoculation, the seedlings were gently removed from the potting mix, the roots of each plant were washed with tap water, and each root was rated for clubroot symptom severity on a 0 to 3 scale, where: 0 = no clubs, 1 = a few small clubs on less than one-third of the roots, 2 = moderate clubs (small to medium-sized clubs on 1/3 to 2/3 of the roots), and 3 = severe clubs (medium to large-sized clubs on > 2/3 of the roots). A disease severity index (DSI) was then calculated using the formula of Horiuchi and Horias, modified by Strelkov et al.^54^.

$$\mathrm{DSI} \left(\%\right)=\frac{\Sigma (n\times 0+n\times 1+n\times 2+n\times 3)}{N\times 3}\times 100$$

where *n* is the number of plants in a class; *N* is the total number of plants in an experimental unit; and 0, 1, 2 and 3 are the symptom severity classes.

Assessment of the disease reaction of each accession with a resistant response (DSI < 20) in the initial test was repeated two more times. Each of these repetitions provided a similar result.

### Sequence analysis and SNP discovery

The accession sequences were analyzed using genotyping by sequencing (GBS) in four major steps; DNA sample preparation, library construction, library sequencing and SNP calling. For sample preparation, DNA extraction was performed using the DNeasy 96 plant kit as per the manufacturer’s instruction (Qiagen). To reduce the genome complexity, DNA was digested with ApeKI, a methylation-sensitive restriction enzyme. For library construction, the fragments produced by digestion were directly ligated to enzyme-specific adapters followed by PCR amplification. For sequencing, the samples were divided into two pools of 96 samples each, and assessed in two runs of Illumina HiSeq 2500 (Illumina Inc., USA). DNA alignment was generated with BWA software version 0.7.8-r455. The GBS-TASSEL pipeline^55^ was used for SNP calling, and VCF and HapMap genotype files were generated. Initial SNP filtration was performed with the following settings: minor allele frequency (MAF) > 0.01 and missing data per site < 90%. Accessions with too much missing data were removed. Depth, missingness and heterozygosity were calculated using VCFtools V.0.1.12^56^. Genotyping and SNP calling were performed at the Genomic Diversity Facility, Cornell University (http://www.bio-tech. cornell.edu/brc /brc/ services). Beagle 5.1^57^ was used to impute missing genotypes.

### Variant analysis and annotation

Variants were annotated to regions of the *B. napus* reference genome v4.1 downloaded at https://wwwdev.genoscope.cns.fr/brassicanapus/data/ using SnpEff^58^, and Variant Effect Predictor (VEP)^59^, and variant locations were characterised as coding, intron, splice site, promoter and intergenic regions, intergenic, upstream, downstream, and synonymous.

### Genetic diversity and population structure

Population-based genetic diversity, including allele frequencies, minor allele frequency (MAF), and average heterozygosity, were computed using TASSEL 5.2.18 software^60^. Polymorphic information content (PIC) values were calculated for SNP markers using the formula (PIC = 1 − (maf^2^ + (1-maf)^2^))-(2maf^2^(1-maf)^2^)), where maf = is the minor allele frequency. The ratio of transitions to transversions was calculated using the Kimura 2-parameter model in MEGA7^61^.

Structure analysis of the accessions was conducted using fastSTRUCTURE v2.2^62^. The admixture model and correlated allele frequency were applied with a burn-in period of 50,000 iterations and 100,000 replications of Markov Chain Monte Carlo (MCMC).

### Linkage disequilibrium (LD) analysis

LD decay across the *B. napus* genome was measured and a correlation matrix of *r*^2^ values was computed between all pairs of polymorphic SNPs with MAF ≥ 5% using the GAPIT V3 package^63^.

### Genome-wide association analysis

Clubroot severity data (disease severity index, DSI) were transformed using rank-based inverse normal transformation using the rntransform function in the GenABEL R Library^64^. Associations were analyzed for all SNP markers with MAF ≥ 5%, and evenly distributed (1 SNP per 100 Kb) using the following models: general linear model (GLM) for naïve and P + Q, and mixed linear models (MLM) for P + K and P + Q + K, where naïve refers to genotypes and phenotypes only, Q is structure and K is kinship. A kinship matrix of the accessions was calculated and principle components analysis was used to account for population structure and accession relatedness. The association analysis was done using TASSEL v.5^60^.

### Candidate resistance genes

Using Blast2Go software^65^, the sequence regions neighboring and within LD of the significant SNPs were searched for candidate genes encoding disease resistance proteins that were potentially responsible for the resistance to each pathotype of *P. brassicae*.

## Supplementary Information

Supplementary Information 1.
