# Supplementary material for: Identification of resistance loci against new pathotypes of Plasmodiophora brassicae in Brassica napus based on genome-wide association mapping
Source: Sci Rep. 2021 Mar 23;11:6599. doi: 10.1038/s41598-021-85836-9 (PMC7987998; doi:10.1038/s41598-021-85836-9)
Supplement: Supplementary file 1 — Supplementary Information 1. [file 41598_2021_85836_MOESM1_ESM.docx]

**Identification of resistance loci against new pathotypes of *Plasmodiophora brassicae* in *Brassica napus* based on genome-wide association mapping**

Abdulsalam Dakouri^1^, Mebarek Lamara^2^, Md. Masud Karim^1^, Jinghe Wang^1^, Qilin Chen^1^, Bruce D. Gossen^1^, Stephen E. Strelkov^3^ Sheau-Fang Hwang^3^, Gary Peng^1^, Fengqun Yu^1^

^1^ Saskatoon Research and Development Centre, Agriculture and Agri-Food Canada, Saskatoon, Canada.

^2^Institut de Recherche sur les Forêts (IRF), Université du Québec en Abitibi-Témiscamingue, 445 boul. de l’Université, Rouyn-Noranda, QC J9X 5E4, Canada.

^3^ Department of Agricultural, Food and Nutritional Science, University of Alberta, Alberta, Canada.

^*^Corresponding author: Fengqun Yu. Email: [Fengqun.yu@canada.ca](mailto:Fengqun.yu@canada.ca)

**Table S1.** List of accessions, their growth habits, type, origin and DSIs

| Accession name | Habit | Type | Origin | Disease severity index (DSI) | | | | | | |
| --- | --- | --- | --- | --- | --- | --- | --- | --- | --- | --- |
|  |  |  |  | 5X- L-G02 | | 2B | | 3A | | 3D |
| Ames1669 | Spring | oilseed rape | North America | 42 | . | | . | | . | |
| Ames20126 | Spring | oilseed rape | North America | 100 | . | | 11.1 | | . | |
| Ames6074 | Spring | oilseed rape | North America | 26 | . | | . | | . | |
| Ames6075 | Spring | oilseed rape | North America | 28 | . | | . | | . | |
| Ames6076 | Spring | oilseed rape | North America | 38 | . | | . | | . | |
| CGN06820 | Winter | oilseed rape | Europe | 94 | 100 | | 86.7 | | 40 | |
| CGN06821 | Winter | oilseed rape | Europe | 94 | 100 | | 100 | | 72.2 | |
| CGN06822 | Winter | oilseed rape | Europe | 0 | 0 | | 5.6 | | 5.6 | |
| CGN06868 | Winter | oilseed rape | Europe | 75 | 100 | | 100 | | 50 | |
| CGN06869 | Winter | oilseed rape | Europe | 97 | 100 | | 33.3 | | 50 | |
| CGN06870 | Winter | oilseed rape | Europe | 97 | 33.3 | | 50 | | 40 | |
| CGN06873 | Winter | oilseed rape | Europe | 97 | 100 | | 100 | | 66.7 | |
| CGN06875 | Winter | oilseed rape | Europe | 100 | 100 | | 100 | | 100 | |
| CGN06876 | Winter | oilseed rape | Europe | 97 | 100 | | 100 | | 41.7 | |
| CGN06879 | Winter | oilseed rape | Europe | 100 | 100 | | 100 | | 91.7 | |
| CGN06883 | Winter | oilseed rape | Europe | 78 | 100 | | 100 | | 100 | |
| CGN06884 | Winter | oilseed rape | Europe | 14 | 100 | | 100 | | 100 | |
| CGN06885 | Winter | oilseed rape | Europe | 78 | 100 | | 100 | | 100 | |
| CGN06887 | Winter | fodder rapa | Oceania | 97 | 78 | | 96 | | 83 | |
| CGN06888 | Winter | fodder rapa | Europe | 92 | 33 | | 100 | | 100 | |
| CGN06890 | Winter | fodder rapa | Europe | 42 | 94.4 | | 100 | | 50 | |
| CGN06893 | Spring | oilseed rape | Europe | 94 | 100 | | 100 | | 100 | |
| CGN06894 | Winter | oilseed rape | Europe | 100 | 100 | | 100 | | 66.7 | |
| CGN06895 | Winter | oilseed rape | Europe | 97 | 100 | | 91.7 | | 42.9 | |
| CGN06896 | Winter | oilseed rape | Europe | 0 | 50 | | 100 | | 50 | |
| CGN06897 | Winter | oilseed rape | Asia | 72 | 100 | | 95.8 | | 85.7 | |
| CGN06898 | Winter | oilseed rape | Europe | 100 | 66.7 | | 100 | | 33.3 | |
| CGN06899 | Winter | Swede rape | Europe | 67 | 67 | | 67 | | 67 | |
| CGN06900 | Winter | Swede rape | Europe | 6 | 100 | | 100 | | 100 | |
| CGN06901 | Winter | Swede rape | Europe | 0 | 0 | | 0 | | 0 | |
| CGN06902 | Winter | Swede rape | Oceania | 97 | 100 | | 100 | | 100 | |
| CGN07227 | Winter | oilseed rape | Europe | 94 | 97 | | 97 | | 97 | |
| CGN07229 | Winter | oilseed rape | Europe | 97 | 100 | | 100 | | 100 | |
| CGN07230 | Winter | fodder rapa | Europe | 100 | 100 | | 100 | | 100 | |
| CGN07231 | Winter | fodder rapa | Europe | 97 | 10 | | 100 | | 100 | |
| CGN07232 | Winter | fodder rapa | Europe | 97 | 97 | | 97 | | 97 | |
| CGN07233 | Winter | fodder rapa | Europe | 97 | 0 | | 16.7 | | 0 | |
| CGN07234 | Winter | fodder rapa | Europe | 100 | 100 | | 100 | | 100 | |
| CGN07235 | Winter | fodder rapa | Europe | 100 | 100 | | 100 | | 100 | |
| CGN07236 | Spring | oilseed rape | Europe | 100 | 100 | | 100 | | 33.3 | |
| CGN07237 | Winter | Swede rape | Europe | 94 | 100 | | 100 | | 100 | |
| CGN07311 | Winter | fodder rapa | Europe | 92 | 92 | | 92 | | 92 | |
| CGN11016 | Winter | oilseed rape | Europe | 100 | 100 | | 100 | | 66.7 | |
| CGN11017 | Winter | oilseed rape | Unknown | 100 | 50 | | 50 | | 75 | |
| CGN12014 | Spring | oilseed rape | Europe | 100 | 94.4 | | 96.7 | | 90 | |
| CGN13907 | Winter | fodder rapa | Europe | 100 | 100 | | 100 | | 83.3 | |
| CGN13908 | Winter | fodder rapa | Europe | 94 | 100 | | 100 | | 91.7 | |
| CGN13910 | Winter | fodder rapa | Europe | 94 | 100 | | 100 | | 76.2 | |
| CGN13911 | Winter | fodder rapa | Europe | 100 | 100 | | 100 | | 50 | |
| CGN13912 | Winter | oilseed rape | Europe | 94 | 100 | | 100 | | 80.6 | |
| CGN13918 | Winter | fodder rapa | Europe | 67 | 66.7 | | 22.2 | | 40 | |
| CGN13920 | Winter | fodder rapa | Europe | 72 | 100 | | 100 | | 44.4 | |
| CGN13921 | Winter | fodder rapa | Europe | 94 | 90.9 | | 100 | | 63.9 | |
| CGN13923 | Winter | fodder rapa | Europe | 92 | 69.7 | | 86.1 | | 88.9 | |
| CGN13934 | Winter | oilseed rape | Europe | 100 | 52.8 | | 66.7 | | 47.2 | |
| CGN14113 | Winter | oilseed rape | Europe | 89 | 89 | | 89 | | 89 | |
| CGN14115 | Winter | oilseed rape | Europe | 97 | 91.7 | | 91.7 | | 11.1 | |
| CGN15177 | Winter | fodder rapa | Europe | 100 | 22.2 | | 33.3 | | 0 | |
| CGN15181 | Winter | fodder rapa | Europe | 94 | 66.7 | | 83.3 | | 66.7 | |
| CGN15182 | Winter | fodder rapa | Europe | 86 | 61.1 | | 11.1 | | 16.7 | |
| CGN17300 | Winter | oilseed rape | Europe | 94 | 100 | | 66.7 | | 51.5 | |
| CGN17302 | Winter | oilseed rape | Europe | 92 | 100 | | 70.8 | | 36.1 | |
| CGN17303 | Winter | oilseed rape | Europe | 83 | 100 | | 100 | | 38.9 | |
| CGN17304 | Winter | oilseed rape | Europe | 100 | 100 | | 66.7 | | 18.2 | |
| CGN17306 | Winter | oilseed rape | Africa | 100 | 100 | | 100 | | 100 | |
| CGN17308 | Winter | oilseed rape | Europe | 100 | 100 | | 100 | | 55.6 | |
| CGN17309 | Winter | oilseed rape | Europe | 92 | 66.7 | | 61.1 | | 80 | |
| CGN17310 | Winter | oilseed rape | Europe | 94 | 80 | | 80.6 | | 75 | |
| CGN17312 | Winter | oilseed rape | Europe | 100 | 95.8 | | 93.9 | | 22.2 | |
| CGN17313 | Winter | oilseed rape | Europe | 89 | 100 | | 100 | | 8.3 | |
| CGN17314 | Winter | oilseed rape | Europe | 94 | 100 | | 100 | | 33.3 | |
| CGN17316 | Winter | oilseed rape | Europe | 94 | 50 | | 77.8 | | 0 | |
| CGN17317 | Winter | oilseed rape | Europe | 100 | 100 | | 100 | | 83.3 | |
| CGN17321 | Winter | oilseed rape | Europe | 97 | 94.4 | | 75 | | 33.3 | |
| CGN17322 | Winter | oilseed rape | Europe | 89 | 83.3 | | 100 | | 33.3 | |
| CGN17323 | Winter | oilseed rape | Europe | 100 | 94.4 | | 100 | | 66.7 | |
| CGN17330 | Winter | oilseed rape | Europe | 94 | 100 | | 93.3 | | 61.1 | |
| CGN17331 | Winter | oilseed rape | Europe | 58 | 100 | | 97 | | 75 | |
| CGN17333 | Winter | oilseed rape | Europe | 83 | 100 | | 100 | | 46.7 | |
| CGN17337 | Winter | oilseed rape | Europe | 97 | 100 | | 100 | | 20 | |
| CGN17339 | Winter | oilseed rape | Europe | 86 | 95.2 | | 91.7 | | 14.8 | |
| CGN17342 | Winter | oilseed rape | Europe | 100 | 83.3 | | 100 | | 76.7 | |
| CGN17344 | Winter | oilseed rape | Europe | 100 | 100 | | 95.8 | | 33.3 | |
| CGN17347 | Winter | oilseed rape | Europe | 100 | 100 | | 100 | | 33.3 | |
| CGN17348 | Winter | oilseed rape | Europe | 100 | 94.4 | | 97 | | 36.7 | |
| CGN17352 | Winter | oilseed rape | Europe | 97 | 100 | | 100 | | 30.3 | |
| CGN17353 | Winter | oilseed rape | Europe | 92 | 90 | | 91.7 | | 66.7 | |
| CGN17358 | Winter | oilseed rape | Europe | 89 | 91.7 | | 97.2 | | 63.9 | |
| CGN17363 | Winter | oilseed rape | Europe | 100 | 100 | | 100 | | 100 | |
| CGN17364 | Winter | oilseed rape | Europe | 100 | 97.2 | | 100 | | 41.7 | |
| CGN17367 | Winter | oilseed rape | Europe | 97 | 96.7 | | 100 | | 91.7 | |
| CGN17369 | Winter | oilseed rape | Europe | 0 | 0 | | 0 | | 0 | |
| CGN17370 | Winter | oilseed rape | Europe | 94 | 94.4 | | 100 | | 33.3 | |
| CGN17371 | Winter | oilseed rape | Europe | 97 | 91.7 | | 100 | | 27.3 | |
| CGN17372 | Winter | oilseed rape | Europe | 81 | 100 | | 100 | | 27.8 | |
| CGN17373 | Winter | oilseed rape | Europe | 80 | 100 | | 100 | | 33.3 | |
| CGN17374 | Winter | oilseed rape | Europe | 77 | 100 | | 100 | | 30.6 | |
| CGN17375 | Winter | oilseed rape | Europe | 100 | 94.4 | | 88.9 | | 44.4 | |
| CGN17376 | Winter | oilseed rape | North America | 83 | 97.2 | | 100 | | 52.8 | |
| CGN17377 | Winter | oilseed rape | Europe | 12 | 69.4 | | 77.8 | | 15.2 | |
| CGN17379 | Winter | oilseed rape | Europe | 14 | 83.3 | | 100 | | 40.7 | |
| CGN17380 | Winter | oilseed rape | Europe | 25 | 91.7 | | 97.2 | | 47.2 | |
| CGN17381 | Winter | oilseed rape | Europe | 0 | 97.2 | | 97.2 | | 30.6 | |
| CGN17382 | Winter | oilseed rape | Europe | 97 | 100 | | 93.3 | | 50 | |
| CGN17383 | Winter | oilseed rape | Europe | 100 | 91.7 | | 94.4 | | 72.2 | |
| CGN18956 | Winter | oilseed rape | Europe | 97 | 100 | | 86.1 | | 83.3 | |
| CGN18957 | Winter | oilseed rape | Europe | 0 | 100 | | 100 | | 40.7 | |
| CGN18960 | Winter | oilseed rape | Europe | 100 | 97.2 | | 100 | | 88.9 | |
| CGN18961 | Winter | oilseed rape | Europe | 94 | 100 | | 100 | | 100 | |
| CGN18962 | Winter | oilseed rape | Europe | 78 | 83.3 | | 83.3 | | 55.6 | |
| CGN18968 | Winter | oilseed rape | Europe | 83 | 97.2 | | 91.7 | | 88.9 | |
| CGN18972 | Winter | oilseed rape | Europe | 89 | 94.4 | | 100 | | 75 | |
| CGN18973 | Winter | oilseed rape | Europe | 83 | 100 | | 100 | | 66.7 | |
| CGN18974 | Winter | oilseed rape | Europe | 97 | 94.4 | | 100 | | 69.4 | |
| CGN18977 | Winter | oilseed rape | Europe | 92 | 97.2 | | 88.9 | | 66.7 | |
| CGN19953 | Winter | oilseed rape | Europe | 69 | 90 | | 100 | | 66.7 | |
| CGN19959 | Winter | oilseed rape | Europe | 86 | 100 | | 100 | | 88.9 | |
| CGN19964 | Spring | oilseed rape | North America | 100 | 91.7 | | 91.7 | | 100 | |
| CGN19966 | Spring | oilseed rape | Asia | 97 | 100 | | 100 | | 85.7 | |
| CGN19967 | Spring | oilseed rape | Europe | 100 | 100 | | 100 | | 66.7 | |
| CN101857 | Spring | oilseed rape | Asia | 77 | 100 | | 75 | | 100 | |
| CN101858 | Spring | oilseed rape | Asia | 63 | 80 | | 100 | | 83.3 | |
| CN101859 | Spring | oilseed rape | Asia | 100 | 94.4 | | 71.4 | | 91.7 | |
| CN101860 | Spring | oilseed rape | Asia | 73 | 83.3 | | 100 | | 70.4 | |
| CN101872 | Spring | oilseed rape | South America | 44 | 100 | | 100 | | 91.7 | |
| CN101873 | Spring | oilseed rape | North America | 61 | 100 | | 100 | | 100 | |
| CN101874 | Spring | oilseed rape | North America | 71 | 100 | | 100 | | 100 | |
| CN101875 | Spring | oilseed rape | North America | 10 | 100 | | 100 | | 75 | |
| CN107671 | Spring | oilseed rape | Asia | 0 | . | | . | | . | |
| CN107681 | Spring | oilseed rape | North America | 0 | 100 | | 100 | | 0 | |
| CN30467 | Winter | turnip | Europe | 100 | 100 | | 88.9 | | 63.9 | |
| CN31149 | Winter | Rutabaga | Europe | 10 | . | | . | | . | |
| CN31150 | Winter | Rutabaga | Europe | 5 | . | | . | | . | |
| CN31734 | Winter | oilseed rape | Europe | 100 | 100 | | 100 | | 100 | |
| CN35453 | Spring | oilseed rape | North America | 100 | 95.8 | | 100 | | 100 | |
| CN35641 | Winter | Swede rape | Europe | 60 | 97.2 | | 88.9 | | 44.4 | |
| CN35644 | Winter | Swede rape | Europe | 100 | 100 | | 100 | | 100 | |
| CN35996 | Winter | oilseed rape | Asia | 100 | 91.7 | | 100 | | 36.1 | |
| CN35997 | Winter | oilseed rape | Asia | 100 | 88.9 | | 94.4 | | 57.6 | |
| CN35998 | Winter | oilseed rape | Asia | 100 | 85.7 | | 91.7 | | 94.4 | |
| CN40224 | Spring | oilseed rape | Europe | 0 | . | | . | | . | |
| CN43442 | Spring | oilseed rape | Asia | 100 | 97.2 | | 86.1 | | 75 | |
| CN43443 | Spring | oilseed rape | Asia | 100 | 100 | | 100 | | 100 | |
| CN43444 | Spring | oilseed rape | Asia | 100 | 91.7 | | 93.3 | | 69.4 | |
| CN43680 | Winter | oilseed rape | Asia | 100 | 100 | | 83.3 | | 45.8 | |
| CN43823 | Spring | oilseed rape | North America | 77 | 100 | | 94.4 | | 100 | |
| CN43824 | Spring | oilseed rape | North America | 100 | 75 | | 100 | | 69.7 | |
| CN45052 | Spring | oilseed rape | Asia | 86 | 100 | | 100 | | 91.7 | |
| CN45053 | Spring | oilseed rape | Asia | 100 | 100 | | 100 | | 100 | |
| CN46234 | Winter | oilseed rape | North America | 10 | . | | . | | . | |
| CN46235 | Spring | oilseed rape | North America | 100 | 100 | | 100 | | 100 | |
| CN46333 | Spring | oilseed rape | North America | 100 | 88.9 | | 100 | | 100 | |
| CN46720 | Spring | oilseed rape | North America | 82 | 85.7 | | 100 | | 75 | |
| CN52741 | Spring | oilseed rape | North America | 100 | 100 | | 100 | | 100 | |
| MENDEL | Winter | oilseed rape | Europe | 0 | 44.4 | | 30.6 | | 27.8 | |
| Pabularia | Winter | oilseed rape | Europe | 0 | . | | . | | . | |
| PI169080 | Winter | oilseed rape | Europe | 27 | 100 | | 100 | | 100 | |
| PI232895 | Winter | oilseed rape | Europe | 88 | 88 | | 88 | | 88 | |
| PI250135 | Spring | oilseed rape | Asia | 97 | 97 | | 97 | | 97 | |
| PI271452 | Spring | oilseed rape | Asia | 100 | 100 | | 100 | | 100 | |
| PI305280 | Winter | oilseed rape | Europe | 43 | 100 | | 55.6 | | 58.3 | |
| PI383422 | Winter | oilseed rape | Europe | 100 | 97.2 | | 100 | | 77.8 | |
| PI399418 | Winter | oilseed rape | Europe | 100 | 100 | | 100 | | 100 | |
| PI431574 | Spring | oilseed rape | North America | 100 | 100 | | 100 | | 100 | |
| PI432391 | Spring | oilseed rape | Asia | 100 | 100 | | 100 | | 100 | |
| PI432393 | Spring | oilseed rape | Asia | 100 | 0 | | 0 | | 0 | |
| PI432395 | Spring | oilseed rape | Asia | 100 | 100 | | 100 | | 87.5 | |
| PI443015 | Winter | oilseed rape | Europe | 3 | 11.1 | | 4.2 | | 28.6 | |
| PI469724 | Spring | oilseed rape | Asia | 100 | 100 | | 100 | | 100 | |
| PI469726 | Spring | oilseed rape | Asia | 100 | 100 | | 100 | | 100 | |
| PI469737 | Spring | oilseed rape | Asia | 100 | 100 | | 100 | | 100 | |
| PI469747 | Spring | oilseed rape | Asia | 100 | 100 | | 100 | | 100 | |
| PI469796 | Winter | oilseed rape | Europe | 100 | 100 | | 100 | | 100 | |
| PI469802 | Spring | oilseed rape | Asia | 100 | 100 | | 100 | | 100 | |
| PI469890 | Spring | oilseed rape | Asia | 0 | . | | . | | . | |
| PI469917 | Spring | oilseed rape | Asia | 100 | 100 | | 100 | | 100 | |
| PI469951 | Spring | oilseed rape | Asia | 100 | 100 | | 100 | | 100 | |

“.” Untested due to poor quality of seed.

b

a

C

D

**Fig. S1.**  Frequency distribution of rank-transformed disease severity index for pathotypes (a) 5X, (b) 2B, (c) 3A, and (d) 3D.


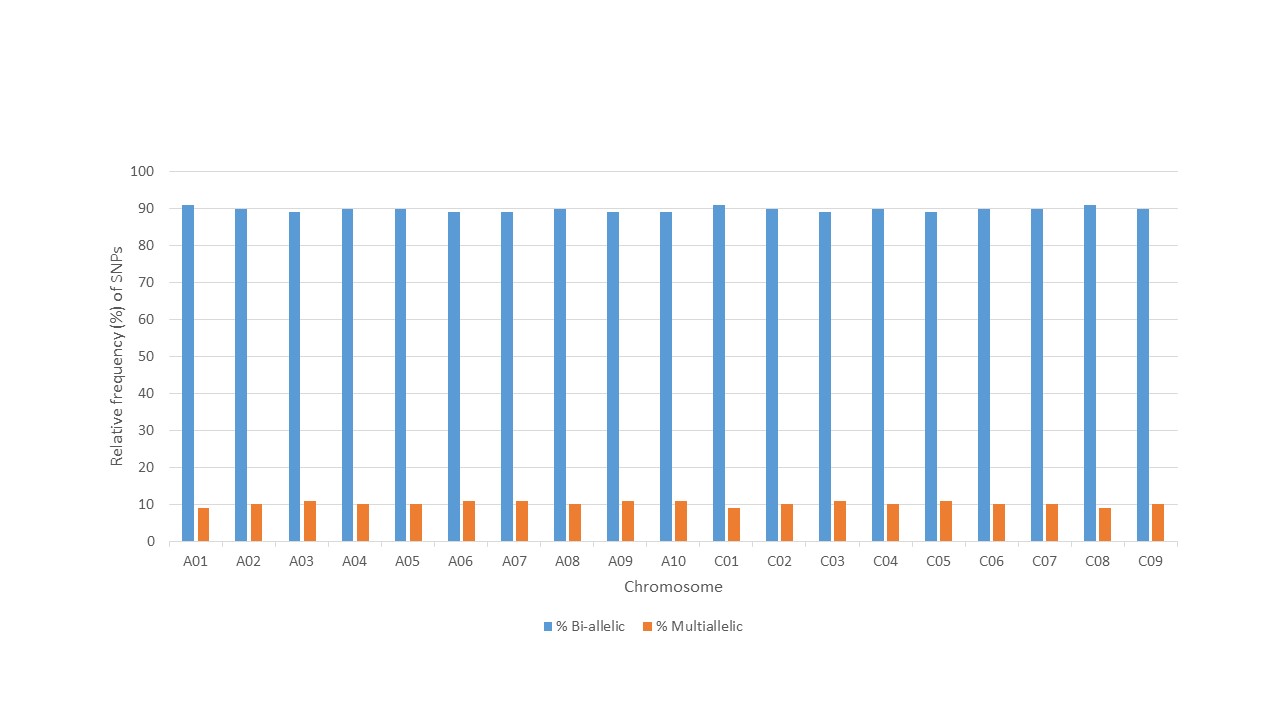


**Fig. S2.** Relative frequency distribution (%) of bi-/multi-allelic SNPs


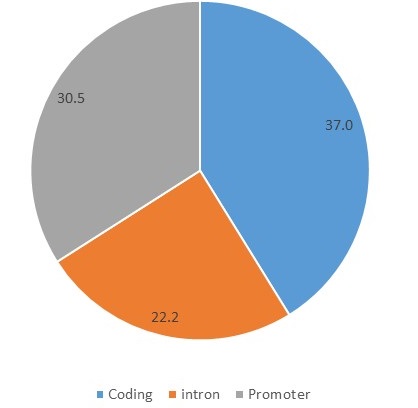


**a**


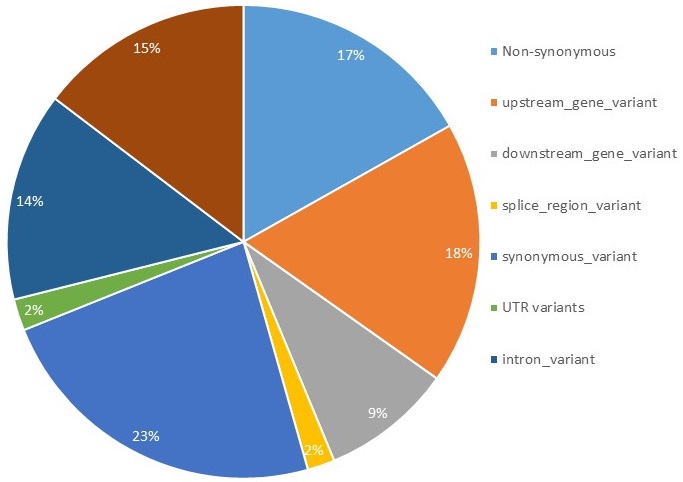


**b**

**c**


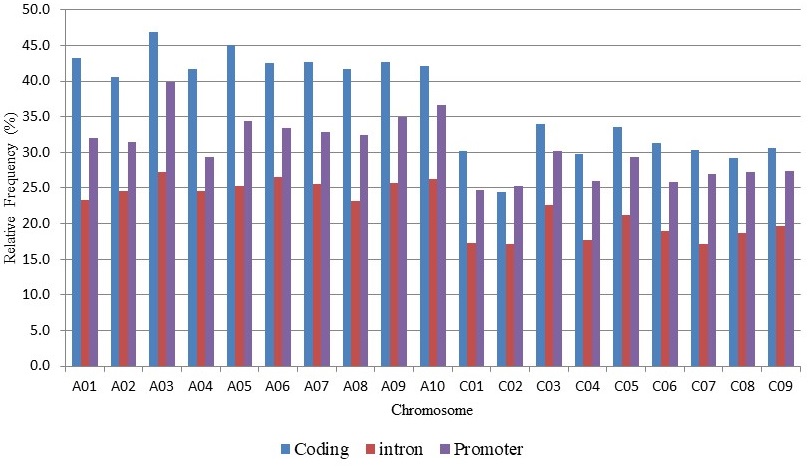


**Fig. S3.** Variant annotation results, **A.** the distribution of SNPs within genic regions; coding region, introns and promotor region, **B**. Derailed SNP annotation based on Variant Effect Predictor software, **C.** Distribution of annotated SNP across *B. napus* chromosomes.


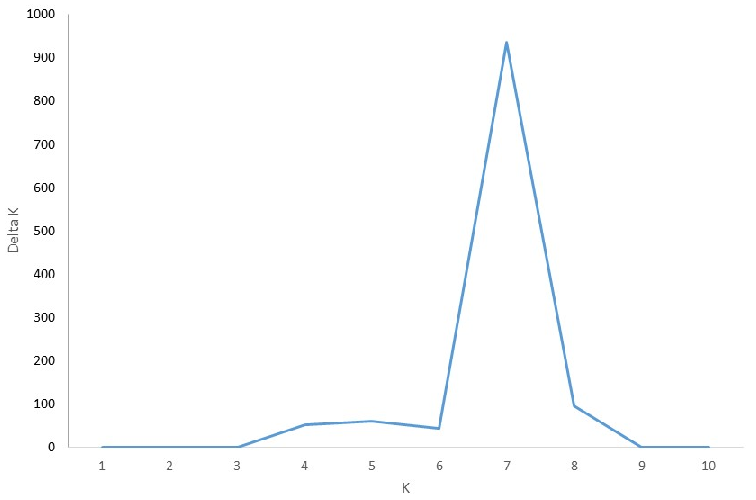


**Fig.S4:** Estimation of the number of populations based on the Evanno criterion.

Ad-hoc statistic delta K for K values of 1 to 10.


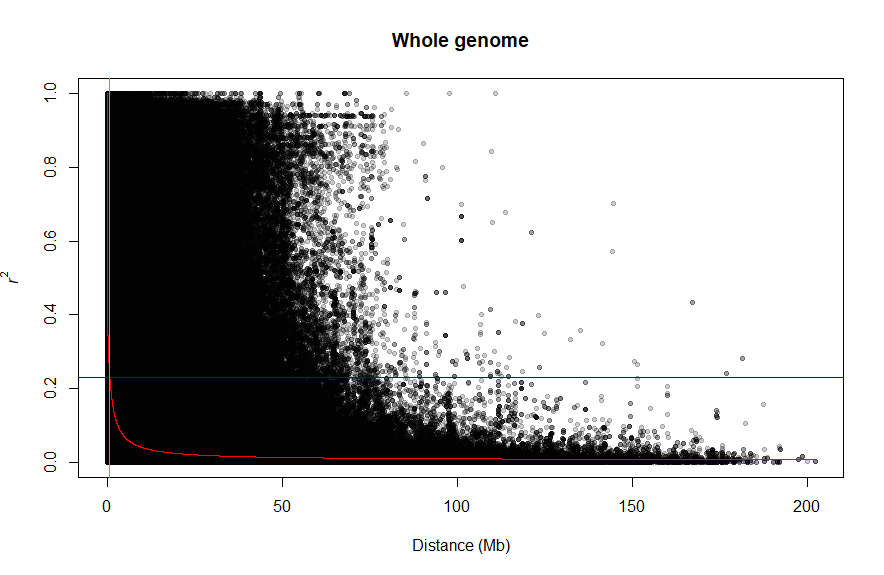


**Fig. S5.** Genome wide linkage disequilibrium (LD) decay plot as a function of physical distance (bp). LD decay assessed in a *B. napus* collection of 177 accessions LD estimates are reported as squared correlations of allele frequencies (r^2^).
